# Inflammatory responses in early pregnancy: Physiological and pathological perspectives

**DOI:** 10.1002/rmb2.12619

**Published:** 2024-12-15

**Authors:** Yasuyuki Negishi, Rimpei Morita

**Affiliations:** ^1^ Department of Microbiology and Immunology Nippon Medical School Tokyo Japan; ^2^ Department of Obstetrics and Gynecology Nippon Medical School Tokyo Japan

**Keywords:** chronic endometritis, endometrial microbiota, inflammation, recurrent pregnancy loss, repeated implantation failure

## Abstract

**Background:**

Several conditions such as infertility, repeated implantation failure, and recurrent pregnancy loss can pose challenges in early pregnancy. These issues can be caused by the abnormal inflammatory response with various factors, including exogenous and endogenous agents, and pathogenic and nonpathogenic agents. In addition, they can be exacerbated by maternal immune response to the abovementioned factors.

**Methods:**

This review aimed to assess the detrimental inflammatory effects of chronic endometritis, endometrial microbiota disturbance, and maternal immune system abnormalities on early pregnancy. Further, essential details such as ovulation, implantation, trophoblast invasion, and placental formation, were examined, thereby highlighting the beneficial roles of inflammation.

**Main Findings:**

Excessive inflammation was associated with various early pregnancy disorders. Meanwhile, a lack of appropriate inflammation could also contribute to the development of different early pregnancy complications.

**Conclusion:**

Excessive inflammation and insufficient inflammation can possibly lead to abnormal conditions in early pregnancy, and appropriate inflammation is required for a successful pregnancy.

## Graphical abstract

Not only excess inflammation but also lack of inflammation can contribute to the development of complications in early pregnancy. Appropriate inflammation is required for successful offspring.

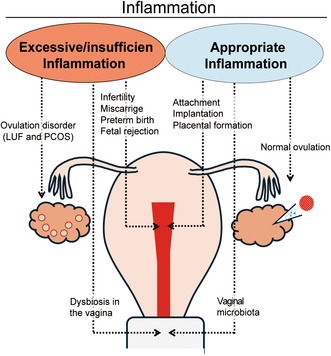

## 1 INTRODUCTION

Inflammation plays an important role in the defense mechanisms of hosts against microbial infections. It includes the phagocytic activity of macrophages and neutrophils during bacterial infections, and the recognition, activation, and cytotoxic functions of T cells and B cells in response to viral infections. These immune cells are essential for eliminating microbes. Further, inflammation that is not involved in pathogen infection is important. Independent of pathogen infection, abnormal, excessive, or suppressed inflammation is commonly observed in various pathologies, including cancer,^1^ cardiovascular diseases,^2^, ^3^, ^4^ diabetic kidney disease,^5^ and pulmonary disorders.^6^ Inflammation without evident pathogenic infection, referred to as sterile inflammation, involves the collaboration of the innate and acquired immune systems to regulate inflammatory reactions. These inflammatory abnormalities are often triggered by exogenous nonpathogenic agents, such as silica, asbestos, and particulate matter 2.5 (PM 2.5).^7^ Thus, inflammation, whether due to infection or not, serves as a biological response that maintains homeostasis in the body in response to various stimuli.

Inflammatory responses are generally triggered by alarmins, which include infection‐related molecules and microparticles. Pathogen‐associated molecular patterns (PAMPs), such as lipopolysaccharide (LPS), peptidoglycan, flagellin, and viral particles, can function as alarmins.^8^, ^9^ These molecules are recognized by the immune system, which then triggers an inflammatory response that alerts the body to the presence of pathogens. As alarmins, PAMPs activate immune cells and initiate a cascade of immune responses, helping in the defense against infections. When cells are damaged, endogenous molecules, such as high‐mobility group box 1 (HMGB1), interleukin (IL)‐1α, IL‐33, heat shock proteins, and S100 proteins, are released into the extracellular space. These molecules, known as damage‐associated molecular patterns (DAMPs), play a critical role in triggering and inducing inflammation. They are also referred to as inflammatory alarmins.^9^ Various factors can trigger the release of DAMPs during pregnancy, including hypoxia, ischemia, vascular dysfunction, and oxidative stress associated with placental insufficiency.^10^ Additional factors include maternal alcohol consumption, smoking, poor nutrition, and trauma. The abovementioned nonpathogenic exogenous nanoparticles, such as asbestos, silica, PM 2.5, microplastics, also act as alarmins. Recently, the concept of resolution‐associated molecular patterns (RAMPs) has gained attention. Proteins such as heart shock protein (HSP)10, HSP27, HSPA5, HSPB5, and phosphatidylserine have been shown to be a type of RAMPs.^11^ While HMGB1 is considered a DAMP, oxidized HMGB1, in which the cysteine of HMGB1 has been oxidized, has anti‐inflammatory properties as a RAMP.^12^ RAMP stimulation can resolve excessive inflammation induced by DAPMs and PAMPs.^13^

Alarmins are detected by pattern recognition receptors (PRRs) such as Toll‐like receptors (TLRs), C‐type lectin receptors, NOD‐like receptors, and receptors for advanced glycation end‐products mainly in antigen‐presenting cells, macrophages, and dendritic cells (DCs).^14^, ^15^, ^16^, ^17^ If these cells detect antigens via their PRRs, they can influence T cells and other effector cells either via direct interaction between the T‐cell receptor and the major histocompatibility complex or via indirect pathways involving cytokine networks. Various inflammatory responses in vivo are induced, starting with the response of alarmins and the PRRs that receive them.

**FIGURE 1:**
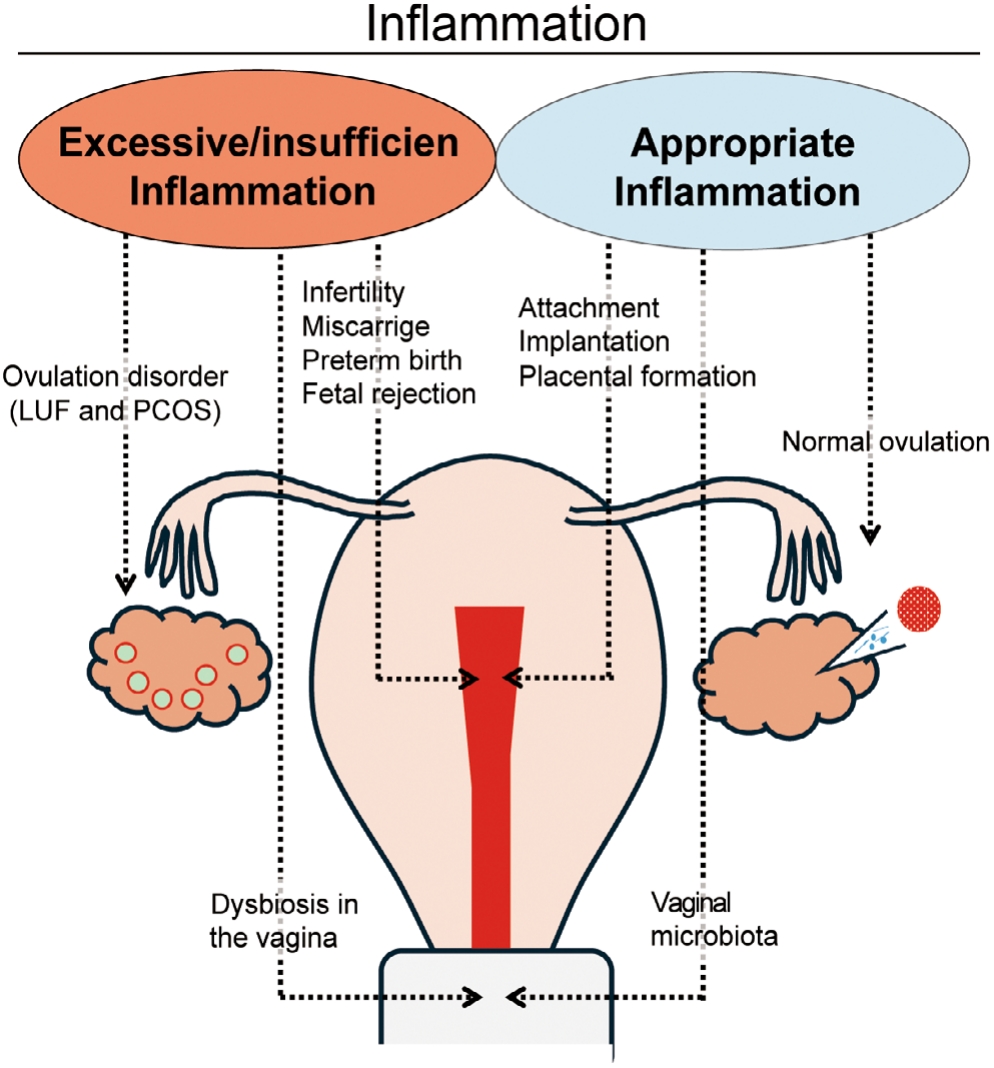
Dual nature of inflammation and its associated pathologies/phenomena during pregnancy. Excessive/insufficient inflammation leads to pregnancy complications. However, an appropriate degree of inflammation is required for a normal pregnancy process.

At the fetal–maternal interface, different immune cells are present. Further, each type of cells is skillfully orchestrated, and they work together to maintain pregnancy. Therefore, if this immune imbalance occurs, various pregnancy complications are triggered. The onset of preterm labor/birth is significantly strongly associated with both pathogenic and nonpathogenic excess inflammation.^18^, ^19^, ^20^, ^21^ Preeclampsia, which is characterized by hypertension, proteinuria, and edema, involves an excessive maternal inflammatory response without microbial infection.^22^, ^23^, ^24^, ^25^ Excessive inflammation can also cause infertility, repeated implantation failure (RIF), recurrent pregnancy loss (RPL), and miscarriage in the early pregnancy period.^26^, ^27^, ^28^ Notably, excessive inflammation induces these pregnancy complications. However, an appropriate level of inflammation is also required for a successful offspring production. Labor onset is caused by uterine inflammation.^29^ Hence, an extremely powerful inflammation is required in late pregnancy and parturition. In early pregnancy, an appropriate level of inflammatory response is required for the attachment and implantation of an embryo, infiltration of trophoblast cells into the maternal tissue, and placentation (Figure 1 and Section 2.2).^30^, ^31^, ^32^ Hence, the retention of a semiallogenic fetus in the maternal body requires the establishment of an anti‐inflammatory environment (i.e., maternal immune tolerance). However, an insufficient inflammation also occasionally contributes to the development of early pregnancy complications (Figure 1 and Section 2.2.3). Further, the mother must have a good immune system to eliminate infectious pathogens to protect the fetus. Thus, during the limited gestation period, the maternal immune system requires prompt alteration in the inflammatory/anti‐inflammatory environment in response to pathological conditions.^33^

This review aimed to assess the beneficial and harmful effects of inflammation in the pathogenesis of various diseases in early pregnancy.

## 2 BENEFICIAL EFFECTS OF INFLAMMATION

### 2.1 Role of inflammation in ovulation

#### 2.1.1 Appropriate inflammation in ovulation

The notion that ovulation involves an inflammatory response was first proposed by Espey^34^ and has since become widely accepted.^35^ Ovulation is triggered by a surge in luteinizing hormone (LH). This surge leads to the invasion of blood vessels into the granulosa cell area and disrupts the basal lamina of these cells, thereby allowing the infiltration of theca cells and leukocytes. After the dilation and increased permeability of these vessels, the cumulus–oocyte complex detaches from the surrounding granulosa cells and expands (cumulus expansion). The follicle eventually deteriorates and ruptures, releasing the cumulus‐enclosed oocyte. Then, the damaged tissue is repaired.

In essence, ovulation represents a complex interaction involving the oocyte, granulosa cells, theca cells, endothelial cells, and both resident and infiltrated immune cells. This process is accompanied by the secretion of various inflammatory mediators, including prostaglandins, reproductive hormones, matrix metalloproteinases, cytokines, and chemokines. The preovulatory follicle contains several inflammatory cytokines such as IL‐1, IL‐2, IL‐6, TNF‐α, granulocyte–macrophage colony‐stimulating factor, and macrophage colony‐stimulating factor.^36^, ^37^, ^38^, ^39^, ^40^, ^41^ The LH surge stimulates the production of chemokines that attract immune cells including neutrophils, monocytes, macrophages, natural killer (NK) cells, B cells, and T cells.^42^, ^43^, ^44^, ^45^, ^46^, ^47^ Macrophages release cytokines and chemokines that help in the migration of other immune cells. Thus, they are essential for follicular growth and rupture.^48^, ^49^, ^50^ Ovarian DCs are important for cumulus expansion, ovulation, and management of ovulation‐related inflammatory response.^51^ Neutrophils, which are recruited by bone morphogenetic protein 6, secrete cytotoxic peptides and proteases that degrade the follicular wall.^52^ NK cells and their chemokine receptors play roles in ovulation and angiogenesis.^42^, ^53^

#### 2.1.2 Inappropriate inflammation in ovulation

Luteinized unruptured follicle (LUF) is a common condition in individuals with infertility. It is characterized by the lack of follicular rupture that results in ovulatory dysfunction.^54^, ^55^, ^56^ LUF may be associated with disruptions in the inflammatory processes involved in ovulation. In humans, the granulocyte‐colony stimulating factor (G‐CSF) levels in the peripheral blood increase during the late follicular phase of a normal ovulatory cycle.^57^ Further, LUF can be alleviated by administering G‐CSF during a clomiphene citrate cycle.^58^, ^59^ In animal models, the systemic depletion of neutrophils leads to a reduced ovulation rate.^60^ These findings indicate that an inappropriate inflammatory response by granulocytes can contribute to LUF development (Figure 1).

Nevertheless, excessive inflammation of course has a harmful effect on ovulation. Polycystic ovary syndrome (PCOS), a major cause of infertility, is associated with endocrine abnormalities, polycystic ovarian morphology, and ovulatory dysfunction.^61^ Further, it is characterized by systemic low‐grade inflammation.^62^, ^63^ Patients with PCOS have elevated levels of C‐reactive protein and inflammatory cytokines.^64^, ^65^, ^66^ The high levels of HMGB1 in adolescents with PCOS can be reduced by anti‐inflammatory treatments such as myo‐inositol and alpha‐lipoic acid.^67^ Excessive inflammatory responses may contribute to the ovulatory dysfunction observed in PCOS (Figure 1). The inflammation involved in ovulation is typically sterile. However, infections caused by pathogens can impair follicular growth. Granulosa cells, which express LPS receptors such as TLR4, CD14, and MD‐2, exhibit disrupted estradiol production and follicular growth failure when exposed to LPS in animal models.^68^, ^69^

### 2.2 Appropriate inflammation in early pregnancy

#### 2.2.1 Appropriate inflammatory cytokine

As mentioned in the previous text, a maternal immune tolerant and anti‐inflammatory environment is essential for retaining a fetus with semi‐allogeneic antigens. However, recent findings have revealed that, in addition to maternal tolerance to fetal allogeneic antigens, an adequate level of inflammation is also required for successful embryo attachment, implantation, and placentation during the early pregnancy period. Previously, inflammation represented by the secretion of IL‐1, IL‐6, IL‐17, interferon gamma (IFN‐γ), and tumor necrosis factor alpha (TNF‐α) is believed to have a harmful effect on the maintenance of pregnancy. However, attachment, implantation, and placentation require adequate levels of these inflammatory cytokines.^30^, ^31^ In humans, proinflammatory responses, such as the secretion of IL‐6, IL‐8, and TNF‐α, are essential for achieving uterine receptivity.^70^, ^71^, ^72^ In addition, increased levels of IL‐12, IL‐1β, TNF‐α, IL‐6, and nitric oxide facilitate embryo attachment to the decidua.^32^ Elevated levels of IFN‐γ, a key Th1 cytokine, are associated with early pregnancy complications.^73^, ^74^, ^75^ Excessive IFN‐γ production caused by increased inflammation can result in fetal rejection. Nevertheless, IFN‐γ also plays a role in modifying uterine vascularization.^76^, ^77^ Further, patients with RPL present with a reduced proportion of IFN‐γ‐ and TNF‐α‐positive cells in the endometrium.^77^ Therefore, IFN‐γ can also have beneficial effects in sustaining a successful pregnancy. Human chorionic gonadotropin, a glycoprotein hormone produced by the placenta, significantly increases during early pregnancy. It stimulates IL‐8 secretion from monocytes and helps in endometrial differentiation and implantation by modulating immune cell activity.^78^, ^79^

#### 2.2.2 Inflammatory role of macrophages and DCs

Macrophages exhibit plasticity, and they are classified into M1 and M2 subtypes, each with different inflammatory effects. M1 macrophages have proinflammatory effects, and M2 macrophages have anti‐inflammatory effects. The presence of M1 macrophages is detrimental to the maintenance of pregnancy. However, emerging evidence shows that M1 macrophages may play an important role in initiating an appropriate inflammatory response during parturition and early pregnancy.^32^ During the implantation period, macrophages are predominantly polarized toward the M1 phenotype, which is associated with the production of proinflammatory cytokines.^71^ After implantation, to help prevent maternal rejection of the fetus as pregnancy progresses, macrophages transition to a mixed M1/M2 phenotype and then to a predominantly M2 phenotype.^32^, ^80^ Toward the end of pregnancy, the re‐emergence of M1 macrophages, which secrete proinflammatory cytokines, becomes essential for parturition.^32^, ^81^

In clinical cases, mechanical injury induced by endometrial biopsy leads to an increased production of macrophage inflammatory protein 3 beta, TNF‐α, CXCL1, osteopontin, and IL‐15, accompanied by an abundance of macrophages and DCs.^82^ The induction of DC and macrophage accumulation via the upregulation of proinflammatory cytokines enhances implantation rates, in vitro fertilization (IVF) outcomes, and clinical pregnancy rates in patients experiencing unexplained infertility.^82^, ^83^, ^84^, ^85^, ^86^ DCs are recruited to the uterus before implantation, and they play an important role in modulating the cytokine profile at the fetal–maternal interface.^87^, ^88^, ^89^, ^90^ Adequate inflammation driven by DCs is essential for successful implantation and for reducing the risk of miscarriage during the first trimester.^91^

Our recent study found reduced levels of DC1s (CD141^+^ DCs), which are responsible for promoting Th1 polarization, in the uterine septum of patients with a septate uterus with low chemokine levels.^92^ Considering that a septate uterus is generally a significant risk factor of RPL, we assumed that a diminished accumulation of DCs with an inflammatory phenotype can result in failed appropriate inflammatory response that creates an immune environment unfavorable for implantation in the uterine septum in early pregnancy.

#### 2.2.3 Approaches according to the endometrial immune profile

Systemic and utero‐local reactions are extremely different; therefore, it is important to investigate the immune profile of the endometrium. The application of various biomarkers is recommended for assessing the immune profile of the human endometrium in patients with RIF.^93^, ^94^ The IL‐18/tumor necrosis factor‐like weak inducer of apoptosis (TWEAK) mRNA ratio is a useful endometrial biomarker of angiogenesis and Th1/Th2 balance during implantation.^93^, ^95^ The IL‐15/fibroblast growth factor‐inducible molecule 14 (Fn‐14) mRNA ratio is a biomarker of uterine NK cell activation and maturation.^93^ Interestingly, neither over‐immune activation (extremely high) nor low‐immune activation (extremely low) that was evaluated using the IL‐18/TWEAK and IL‐15/Fn‐14 ratio is favorable for pregnancy outcomes. Hence, individualized treatment based on the patient's endometrial condition is required.

## 3 HARMFUL EFFECTS OF INFLAMMATION

### 3.1 Chronic endometritis (CE)

The concept of CE is now commonly recognized in clinical practice. CE is defined as inflammation of the endometrium. Clinically, patients with CE are usually asymptomatic or present only subtle symptoms.^96^ However, previous studies have revealed that CE is associated with implantation failure and pregnancy loss.^97^, ^98^, ^99^, ^100^ The incidence rates of CE in patients with infertility and RPL are approximately 2.8%–39% and 60%, respectively.^101^ CE induces endometrial dysfunction and reduces the endometrial receptivity of embryos. Hysteroscopic examinations typically detect stromal edema, thickening of the uterine lining, micropolyps, and focal or diffuse hyperemia in affected patients.^98^, ^102^, ^103^ The pathological feature of CE is the infiltration of CD138 (syndecan‐1)‐positive plasma cells into the endometrial tissue.^98^, ^104^, ^105^, ^106^, ^107^ Classically, CE is diagnosed by identifying the presence of CD138‐positive plasma cells in the endometrial stroma through immunohistochemical staining for CD138^108^; however, this finding is not specific, and its effectiveness can vary depending on the menstrual cycle. Given these limitations, complementary methods such as hysteroscopy and microbial culture are frequently used.^101^ Bacterial infections, such as *Escherichia coli*, *Streptococcus* spp., *Staphylococcus* spp., *Enterococcus faecalis*, *Corynebacterium*, *Mycoplasma*, and *Ureaplasma*, are major causes of CE.^98^, ^103^ Oral antibiotic therapy is the first‐line treatment for CE in patients with pregnancy complications. Notably, antibiotic therapy has been effective in improving implantation, clinical pregnancy, and live birth rates.^102^, ^109^ This finding supports the role of microbial infection in the pathogenesis of CE. However, the most frequently detected infectious agents at the endometrial level are common bacteria, such as *E. coli*, *Streptococcus* spp., and *Staphylococcus* spp.^103^ A previous study evaluated the presence of various pathogens in patients with infertility, with or without CE, using reverse‐transcription polymerase chain reaction. No significant differences were found in the percentage of these pathogens between patients with and without CE.^101^ These results indicate that no specific pathogen is responsible for CE development. In general, the dominance of *Lactobacillus* in the uterus indicates a healthy status (Section 3.2). However, patients with CE had a higher detection rate of *Lactobacillus* than healthy women.^110^ According to these findings, the pathogenesis of CE may involve not only a direct attack by pathogens on the endometrium and embryo but also an abnormal interaction between these pathogens and the immune system in the endometrium. Several theories regarding the immune response related to CE have been proposed. Patients with CE and RIF presented with an abnormal distribution of a NK cell subsets,^111^ and patients with CE exhibited elevated levels of IL‐6 and TNF‐α in menstrual effluents.^112^ Other studies have reported that autophagy dysregulation promotes the production of proinflammatory cytokines and Th17‐dominant milieu in patients with CE^113^ and the accumulation of Th1 cells surrounding CD138‐positive cells in patients with CE.^114^ Abnormalities in maternal immune responses toward pathogens may contribute to reproductive failure.

Patients often do not respond to various antibiotic regimens, indicating the involvement of additional factors. Reports indicate that infections caused by viruses, such as herpes simplex virus,^115^, ^116^ Epstein–Barr virus,^117^ and human immunodeficiency virus,^118^, ^119^ are correlated with CE. Furthermore, other nonpathogenic exogenous agents and endogenous alarmins related to immune disorders may also contribute to CE development.

**FIGURE 2:**
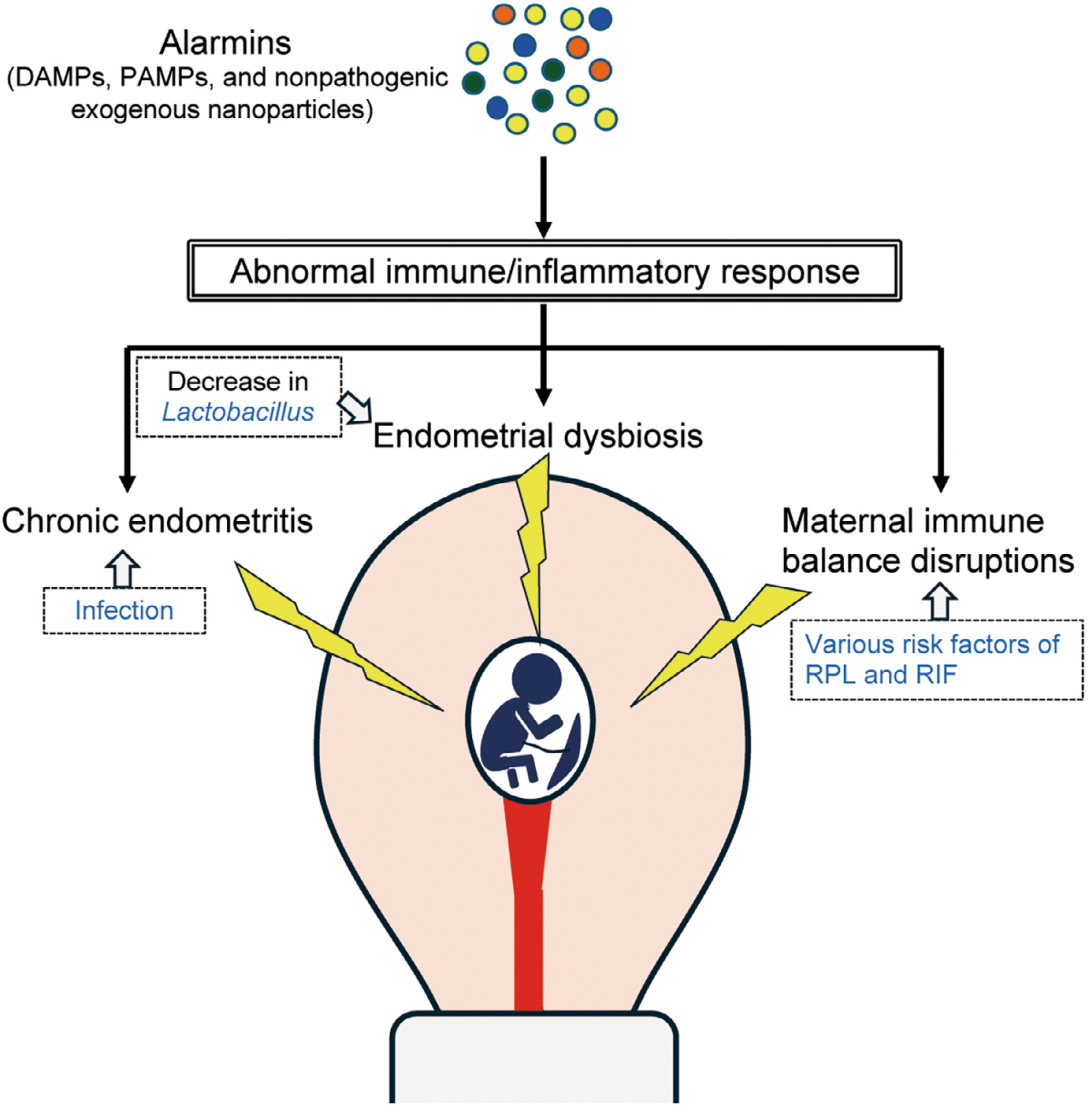
Schematic drawing of complications in early pregnancy via abnormal immune/inflammatory responses. Abnormal immune/inflammatory responses are involved in early pregnancy complications such as chronic endometritis, endometrial dysbiosis, and maternal immune balance disruptions. Alarmins may be deeply involved in triggering these immune/inflammatory abnormalities.

Collectively, pathogens and the abnormal immune response of the endometrium induced by these pathogens can be responsible for CE development (Figure 2). Further detailed analysis may be required to achieve a more precise understanding of the pathogenesis of CE.

### 3.2 Endometrial microbiota disturbance

Previously, the uterine cavity is considered a sterile organ under normal conditions. Next‐generation sequencing of the 16S rRNA gene (16S analysis) is widely used to analyze the microbiota in the reproductive tract. Thus, there is now a consensus that the uterus is not a sterile environment.^120^, ^121^, ^122^ The endometrial microbiota, in numerous bacterial species are present, significantly affect pregnancy outcomes. The *Lactobacillus* dominant microbiota is associated with favorable pregnancy outcomes, pregnancy rate, implantation rate, and live birth rate. Meanwhile, the low abundance of endometrial *Lactobacillus* is associated with poor reproductive outcomes.^123^, ^124^, ^125^ Thus, investigating the endometrial microbiota has become a more frequent component of infertility evaluations. However, the notion that *Lactobacillus* dominance is beneficial for pregnancy outcomes remains controversial. The pregnancy outcomes, pregnancy, and implantation or miscarriage rates did not significantly differ in patients undergoing IVF between the *Lactobacillus* dominant (>80% *Lactobacillus*) and *Lactobacillus* nondominant (<80% *Lactobacillus*) groups.^126^ Moreover, pregnancies are maintained even if the percentage of *Lactobacillus* is extremely low in some cases.^126^ However, improving intrauterine dysbiosis, which is an environment with low abundance of *Lactobacillus*, with antibiotics and prebiotic and probiotic preparations did not improve pregnancy outcomes.^123^ As mentioned in Section 3.1, patients with CE who are likely to present with poor pregnancy outcomes have higher *Lactobacillus* detection rates.^110^ Given these conflicting reports, further investigations are needed to understand the effect of *Lactobacillus* on pregnancy outcomes.

*Lactobacillus* genus contains some species, *L. crispatus*, *L. gasseris*, *L. iners*, and *L. jensenii*. A recent in vitro study revealed that *L. crispatus* promotes trophoblast invasion into the maternal myometrium.^127^ *L. iners* is the most common microbe detected in the endometrium during early pregnancy, and its presence is associated with defense mechanisms and essential functions.^125^ However, a study reported that individuals with *L. iners* as the dominant microbe had the lowest implantation rate after IVF.^128^ *Lactobacillus* produce different isomers of lactic acid. *L. crispatus* and *L. gasseri* produce both d‐lactic acid and l‐lactic acid; however, *L. iners* produces only l‐lactic acid, whereas *L. jensenii* produces only d‐lactic acid.^129^ Importantly, these lactic acids have different acidity and resistance to bacterial infections. *L. crispatus* induces a lower pH in the vaginal environment and has a strong antibacterial effect on *Gardneralla*, which causes bacterial vaginosis. These structural differences in lactic acid may induce different immune responses and contribute to changes in the vaginal and uterine environment. To prove the effectiveness of *Lactobacillus*, the characteristics of pregnancy outcomes must be studied for each of these species.

The uterine cavity contains various innate and acquired immune cells that recognize these bacteria and trigger diverse immune responses. Thus, even with the same bacterial species, differences in individual immune responses are expected (Figure 1). Therefore, pregnancy outcomes may differ even with the same endometrial microbiota. Further investigations should be performed explore the correlation between endometrial dysbiosis and abnormal immune responses, CE, and decidualization and invasion disruptions.

### 3.3 Immunological disorder

A successful pregnancy requires a delicate balance in the maternal immune system (i.e., the mother's immune tolerance) to the paternal semi‐alloantigens of the fetus.^130^, ^131^ Disruption of this maternal tolerance can lead to infertility, implantation failure, miscarriage, and premature birth. Thus, maintaining an appropriate balance between Th1 and Th2 cells is crucial during pregnancy.^132^, ^133^, ^134^, ^135^, ^136^ Recently, the balance between Th17 and regulatory T (Treg) cells, the Th1/Th2/Th17/Treg paradigm, the cytotoxic potential of NK cells, and the involvement of various cytokines have also been identified as important factors.^137^, ^138^, ^139^, ^140^, ^141^, ^142^, ^143^ Patients experiencing complications, such as infertility, implantation failure, and early miscarriage often exhibit excessive inflammation characterized by Th1 dominance, Th17 dominance, and elevated NK cell cytotoxicity. Therefore, accurate diagnosis and treatment for these conditions during early pregnancy are essential. To obtain a diagnosis, the Th1/Th2 ratios, Th17/Treg ratios, NK cell activation, immune profiles of the endometrium, and levels of various autoantibodies in peripheral blood must be evaluated (Section 2.2.3).^94^

In recent years, various immunotherapeutic approaches aimed at inhibiting excessive inflammation in early pregnancy complications have been proposed. In clinical cases, heparin is used in patients with RPL associated with antiphospholipid antibody syndrome. Some studies have indicated that heparin improves live birth rates in cases of RIF.^144^, ^145^ Steroid hormones possess powerful anti‐inflammatory and immunosuppressive effects and can induce an increase in Treg expression,^144^ decrease in uNK cell expression,^146^ and increase in HLA‐G expression in the trophoblasts.^147^, ^148^ Intravenous immunoglobulin G (IVIg) has powerful immunomodulating functions. IVIg can decrease the frequency and activity of NK cells, antibody production of B cells, and expression of activator receptors in monocytes. In contrast, IVIg can increase the expansion and suppressive function of Tregs.^149^ Thus, IVIg has a powerful anti‐inflammatory effect. Recent studies have revealed that high‐dose IVIg administration in early pregnancy improves pregnancy outcomes in women with four or more RPL of unexplained etiology.^150^ Intralipid, a fat emulsion containing soyabean oil, glycerol, and egg phospholipids, inhibits NK cell activity and has been found to be effective in patients with reproductive failure.^151^, ^152^ Additionally, tacrolimus, a major immunosuppressive agent used to prevent organ transplant rejection, has been shown to improve pregnancy outcomes in patients with RIF who present with high Th1/Th2 ratios.^153^, ^154^, ^155^

As outlined, inflammation during pregnancy can have beneficial and harmful effects, and excessive inflammation and insufficient inflammation are risk factors for pregnancy complications. Table 1 shows some representative studies on inflammation and immune function related to early pregnancy, focusing on beneficial/harmful inflammation and immune reaction.

**TABLE 1:** Representative studies on inflammation and immune function related to early pregnancy.

Disease/pathology	Author/years	Description	Roles of inflammation/findings
Ovulation	Duffy (review)/2019^35^	LH^a^ surge and inflammatory response	Beneficial inflammation
	Buyalos/1992^37^	IL‐6 level in the follicular fluid	Beneficial inflammation
	Wang/1992^38^	TNFα in the follicular fluid	Beneficial inflammation
	Nishimura/1998^41^	M‐CSF in the follicular fluid	Beneficial inflammation
	Al‐Alem/2015^42^	Leukocytes and chemokines	Beneficial inflammation
	Kryczek/2005^46^	Chemokine and T cells	Beneficial inflammation
	Nio‐Kobayashi/2015^47^	Chemokine and macrophages	Beneficial inflammation
	Cohen‐Fredarow/2014^51^	Dendritic cells	Both pro‐ and anti‐inflammatory effects
LUF^b^	Shibata/2016^59^	Administration of G‐CSF	Beneficial effect of inflammation for preventing LUF
	Brannstrom/1995^60^	Neutrophils	Beneficial effect of inflammation for preventing LUF
PCOS^c^	Escobar‐Morreale (review)/2011^63^	C‐reactive protein	Harmful inflammation
	Cirillo/2020^67^	HMGB1^d^	Harmful inflammation induced by HMGB1
Placenta formation/fetal growth	Griffith/2017^31^	Inflammation at attachment	Beneficial inflammation at attachment of embryo
	Granot (review)/2012^70^	Inflammatory cytokines and Th1 inflammatory response in implantation	Beneficial inflammation in implantation
	Ashkar/2000^76^	IFN‐γ in placental formation	Beneficial role of IFN‐γ
	Valero‐Pacheco/2022^166^	IL‐33 in placental formation	Beneficial role of IL‐33 in placenta formation and fetal growth
CE^e^	Matteo/2009^111^	Abnormal distribution of NK cell subsets	Harmful inflammation
	Tortorella/2014^112^	Elevation of IL‐6 and TNF‐α levels	Harmful inflammation
	Wang/2019^113^	Autophagy dysregulation and Th17 dominancy	Harmful effects of Th17 dominanct in CE
	Kitazawa/2021^114^	Accumulation of Th1 cells	Harmful inflammation
Endometrial microbiota disturbance	Moreno/2016^124^	Dominancy of *Lactobacillus*	Beneficial effects of *Lactobacillus*
	Hashimoto/2019^126^	Eubiosis and dysbiosis related to *Lactobacillus*	No differences between eubiosis and dysbiosis for implantation
	Yoshida/2021^127^	*L. crispatus*	Beneficial role of *L. crispatus* in trophoblast invasion
	Kadogami/2023^128^	*L. iners*	Low implantation rate in *L. iners* dominant state
Immunological disorder	Saito (review)/2010^137^	Th1/Th2/Th17/Treg^f^ balance	Pregnancy complications and disruption of immune balance
	Fukui/2008^143^	NK cell cytotoxicity	Harmful inflammation in RIF^g^
	Ledee (review)/2016^93^	IL‐18/TWEAK^h^ and IL‐15/Fn‐14^i^ balance	Importance of appropriate balance

^a^ LH, luteinizing hormone.

^b^ LUF, luteinized unruptured follicle.

^c^ PCOS, polycystic ovary syndrome.

^d^ HMGB1, high‐mobility group box 1.

^e^ CE, chronic endometritis.

^f^ Treg, regulatory T.

^g^ RIF, repeated implantation failure.

^h^ TWEAK, tumor necrosis factor‐like weak inducer of apoptosis.

^i^ Fn, fibroblast growth factor‐inducible molecule 14.

The occurrence of disruptions in maternal immune balance remains unclear. The disruptions of maternal immune balance may cause sterile inflammation induced by DAPMs, endometriosis, environmental factors, metabolic disorders, and pathogen‐associated inflammation induced by CE and PAMPs (Figure 2 and Section 3.4). Maternal immune abnormalities caused by these alarmins may be associated with early pregnancy complications, necessitating further elucidation of the efficacy of the abovementioned therapies and their therapeutic targets.

### 3.4 Alarmins and complications in early pregnancy

Some abnormal immune responses are expectedly involved in CE development, endometrial microbiota disturbances, and immunological disorders. However, the initial triggering substances for these abnormal immune responses remain unknown. Stimulations by alarmins, PAMPs and DAPMs, are thought to be significant candidates involved in initiating these abnormal immune responses. TLRs, which recognize PAMPs derived from pathogens during bacterial infections such as LPS, are expressed not only in maternal immune cells but also in trophoblasts and play an important role in miscarriage and early placental formation.^156^, ^157^ Given the involvement of bacterial kinetics in CE onset and endometrial microbiota disturbances, PAMPs derived from bacteria are likely responsible for these complications. Viral infections may also be involved in pregnancy complications. Trophoblasts express TLR3, which recognizes dsRNA, a PAMP of viral origin, and may contribute to pregnancy complications.^158^ DAMPs, which are nonpathogenic, can also cause pregnancy complications.^10^ HMGB1, one of the DAMPs, is released from damaged cells to induce inflammation. Several reports have suggested that an increase in HMGB1 negatively affects the early stages of pregnancy and is associated with miscarriages and implantation failure.^159^, ^160^ Additionally, the S100A8 protein, which is a calcium‐binding protein with a molecular weight of 10–13 kDa having two EF‐hand motifs, is present at high levels in the serum of patients with recurrent miscarriage.^161^ These DAMPs may contribute to maternal immunological disorders that are unrelated to pathogen infections. DAMPs can also have beneficial effects on pregnancy outcomes. For instance, IL‐33, an IL‐1 family cytokine, is a representative DAMP associated with positive pregnancy outcomes.^162^, ^163^, ^164^, ^165^ A recent study revealed that appropriate maternal IL‐33 secretion contributes to placental formation in early pregnancy, leading to healthy fetal growth.^166^ This suggests that certain DAMPs induce appropriate inflammation during pregnancy.

CE, endometrial microbiota disturbance, and maternal immune abnormalities induce excessive inflammation, which can lead to various pregnancy complications. Along with the direct stimulation of alarmins, infections, and the various risk factors of RPL and RIF, abnormal immune responses between the mother and fetus can be involved in this phenomenon (Figure 2).

## 4 CONCLUSIONS

Anti‐inflammatory effects represented by maternal immune tolerance and an appropriate level of proinflammatory response are essential for maintaining pregnancy and ensuring successful offspring. Therefore, rather than using a binary model of inflammation versus anti‐inflammation, the specific functions of various cytokines and immune cells should be elucidated in detail.

## CONFLICT OF INTEREST STATEMENT

All authors declare no conflict of interest for this article.
